# Comparison and verification of detection accuracy for late deceleration with and without uterine contractions signals using convolutional neural networks

**DOI:** 10.3389/fphys.2025.1525266

**Published:** 2025-01-23

**Authors:** Ikumi Sato, Yuta Hirono, Eiri Shima, Hiroto Yamamoto, Kousuke Yoshihara, Chiharu Kai, Akifumi Yoshida, Fumikage Uchida, Naoki Kodama, Satoshi Kasai

**Affiliations:** ^1^ Department of Nursing, Faculty of Nursing, Niigata University of Health and Welfare, Niigata, Japan; ^2^ Major in Health and Welfare, Graduate School of Niigata University of Health and Welfare, Niigata, Japan; ^3^ TOITU Co., Ltd., Tokyo, Japan; ^4^ Department of Obstetrics and Gynecology, Niigata University Graduate School of Medical and Dental Sciences, Niigata, Japan; ^5^ Department of Radiological Technology, Faculty of Medical Technology, Niigata University of Health and Welfare, Niigata, Japan

**Keywords:** cardiotocography, fetal heart rate, late deceleration, nonreassuring fetal status, convolutional neural network

## Abstract

**Introduction:**

Cardiotocography (CTG) is used to monitor and evaluate fetal health by recording the fetal heart rate (FHR) and uterine contractions (UC) over time. Among these, the detection of late deceleration (LD), the early marker of fetal mild hypoxemia, is important, and the temporal relationship between FHR and UC is an essential factor in deciphering it. However, there is a problem with UC signals generally tending to have poor signal quality due to defects in installation or obesity in pregnant women. Since obstetricians evaluate potential LD signals only from the FHR signal when the UC signal quality is poor, we hypothesized that LD could be detected by capturing the morphological features of the FHR signal using Artificial Intelligence (AI). Therefore, this study compares models using FHR only (FHR-only model) and FHR with UC (FHR + UC model) constructed using a Convolutional Neural Network (CNN) to examine whether LD could be detected using only the FHR signal.

**Methods:**

The data used to construct the CNN model were obtained from the publicly available CTU-UHB database. We used 86 cases with LDs and 440 cases without LDs from the database, confirmed by expert obstetricians.

**Results:**

The results showed high accuracy with an area under the curve (AUC) of 0.896 for the FHR-only model and 0.928 for the FHR + UC model. Furthermore, in a validation using 23 cases in which obstetricians judged that the UC signals were poor and the FHR signal had an LD-like morphology, the FHR-only model achieved an AUC of 0.867.

**Conclusion:**

This indicates that using only the FHR signal as input to the CNN could detect LDs and potential LDs with high accuracy. These results are expected to improve fetal outcomes by promptly alerting obstetric healthcare providers to signs of nonreassuring fetal status, even when the UC signal quality is poor, and encouraging them to monitor closely and prepare for emergency delivery.

## 1 Introduction

Cardiotocography (CTG) is the most common technique used to noninvasively record fetal heart rate (FHR) and uterine contraction (UC) over time to monitor and assess fetal health (Pinas and Chandraharan, 2016; Santo et al., 2017). CTG is used to determine the presence or absence of fetal heart deceleration and its type based on the temporal characteristics of the FHR and UC (Lear et al., 2016). One of the critical clinical prognostic signs of fetal outcome is late deceleration (LD), which is caused by a decrease in uteroplacental blood flow associated with uterine contraction, resulting in reduced gas exchange in the placenta. LD has been shown to represent a chemoreceptor-mediated response to fetal hypoxemia. In other words, LD occurs when fetal oxygenation becomes inadequate and the chemoreceptor threshold is exceeded. Consequently, the fetal heart rate gradually decreases and then gradually recovers (Itskovitz et al., 1982; Pinas and Chandraharan, 2016). Occasional LD occurring at less than 50% of uterine contractions has been previously described as an “early marker of fetal mild hypoxemia” (Sameshima et al., 2004), obstetricians should be informed as soon as possible about the occurrence of LD. Early detection of LD is critical because it can prompt close monitoring and preparation for emergency delivery. LD is evaluated visually by obstetricians according to guidelines such as the International Federation of Gynecology and Obstetrics (FIGO) and the Japanese Society of Obstetrics and Gynecology. While the FIGO defines LD as an amplitude of ≥15 bpm, the guidelines for obstetric practice in Japan define mild LD as an amplitude of <15 bpm and severe LD as an amplitude of ≥15 bpm; there are slight differences in the definitions between the guidelines.

The temporal relationship between FHR and UC is an important factor in determining LD; however, the signal quality of UC tends to be lower than that of FHR. External tocodynamometry measures uterine contraction using pressure changes obtained from a sensor attached to the maternal abdominal wall, and studies have indicated that the quality of UC is degraded when there is a large amount of abdominal fat or when the sensor is displaced by the mother’s body movements (Euliano et al., 2013; Vlemminx et al., 2017; Petrozziello et al., 2018). When the quality of UC signals is degraded owing to these causes, a situation arises in which the temporal relationship between FHR and UC, an important factor in determining LD, cannot be determined.

It is crucial that healthcare professionals identify the onset of LD as early as possible. Therefore, in clinical settings, when the quality of the UC signal is poor, attention is paid to the FHR signal pattern, and LD is evaluated based on the FHR signal alone. The morphological characteristics of LD include deceleration with a gradual onset, gradual return to baseline, and/or reduced variability within the deceleration (Ayres-de-Campos et al., 2015). Therefore, obstetricians may extract possible LD signals by capturing the morphological features of the FHR.

To detect the occurrence of LD more quickly without missing it, several efforts are underway to detect LD by automatically analyzing CTGs. Products using signal analysis to assist obstetricians in visual assessment include Omniview-SisPorto^Ⓡ^ 3.5 (Speculum, Lisbon, Portugal) and Trium CTG Online^Ⓡ^ (GE HealthcareVR, Little Chalfont, UK) (Magawa et al., 2021; Bernardes, 2023). Omniview-SisPorto^Ⓡ^ 3.5 is built on signal analysis algorithms compliant with FIGO guidelines, while Trium CTG Online^®^, which is available in Japan, is designed based on the guidelines from the Japanese Society of Obstetrics and Gynecology. In a study comparing the analysis results of each product with visual evaluation by obstetricians, Costa MA et al. evaluated Omniview-SisPorto^Ⓡ^ 3.5 and reported a 68% agreement rate for decelerations (Costa et al., 2010). Contrastingly, Magawa S et al. evaluated Trium CTG Online^Ⓡ^ and reported a sensitivity of 0.93 and specificity of 0.99 for Severe LD (Magawa et al., 2021). A recent study examined the use of machine learning (ML) and deep learning (DL) to detect LD and compared the ML and DL methods (Das et al., 2023). This study reported an improved accuracy when labels determined using fuzzy logic were used as the gold standard. However, when the labels determined by the obstetricians were used as inputs for DL, the receiver operating characteristic (ROC) for LD detection was <0.60.

Although studies using signal analysis, ML, and DL have achieved notable results because both FHR and UC signals were used for analysis, it is thought that if the quality of the UC signal deteriorates, it would not be possible to determine the LD accurately. If LD can be detected when the UC signal is poor, it would be of great clinical significance because it would allow the early detection of LD without missing its onset. We also believe that more advanced signal analysis methods are needed to distinguish between LD and other conditions assessed using only FHR signals. Therefore, we constructed a method for detecting LD using convolutional neural networks (CNNs), which is one of the DL methods that has attracted attention in recent years in the field of obstetrics for research on evaluating the health of fetuses from CTG signals (Arain et al., 2023; Cömert et al., 2019; Hirono et al., 2024a; Ogasawara et al., 2021). Therefore, the objective of this study was to construct a robust model for detecting LD, even when the quality of the UC signal is poor, using a CNN to detect LD from the FHR signal only. The developed Artificial Intelligence (AI) model will enable faster and more accurate detection of LD and improve fetal prognosis.

## 2 Materials and methods

This study was approved by The Institutional Review Board of the Niigata University of Health and Welfare (Approval No. 19397-241014). This section describes the experimental environment, data details, artificial intelligence (AI) models, and evaluation methods.

### 2.1 Materials

The experimental environment was a computer with 128 GB of main memory and an NVIDIA GeForce RTX 4090 GPU (NVIDIA Corporation, Santa Clara, California, U.S.A.).

#### 2.1.1 Database used to detect LD

This study used CTU-UHB data obtained from PhysioNet (Chudáček et al., 2014). CTU-UHB consisted of 552 intrapartum CTG data acquired from 2010 to 2012 in the maternity ward of the University Hospital in Brno, Czech Republic. These data were acquired using STAN S21/S31 (Neoventa Medical, Mölndal, Sweden) and Avalon FM40/FM50 (Philips Healthcare, Amsterdam, Netherlands) electronic fetal monitoring devices; CTG recording started 90 min before delivery, with each data point being up to 90 min long. Each CTG dataset contained FHR and UC signals sampled at 4 Hz. Table 1 lists the statistical properties of the CTU-UHB database, including demographic information and high-risk indicators of the patient population.

**TABLE 1:** Statistical properties of the CTU-UHB database.

Information	Mean	Min	Max
Mother’s age (years)	29.6	18	46
Gravidity	1.4	1	11
Parity	0.4	0	7
Diabetes, *n*	No = 515, Yes = 37		
Hypertension, *n*	No = 508, Yes = 44		
Gestational age (weeks)	40	37	43
Neonate’s weight (g)	3,400	1,970	4,750
PH	7.23	6.85	7.47
BE	−6.38	−26.8	−0.20
BDecf (mmol/L)	4.6	−3.40	26.11
Apgar 1 min	8.26	1	10
Apgar 5 min	9.06	4	10
Neonate’s sex, *n*	Male = 286, Female = 266		
Delivery type, *n*	Vaginal = 506, Cesarean section = 46		

Abbreviations: BE, base excess; BDecf, base deficit extracellular fluid.

#### 2.1.2 Ground truth labeling and data selection

As the LDs of interest in this study were the early marker of fetal mild hypoxemia (Sameshima et al., 2004), we focused on the first LDs that occurred during recording because we considered it necessary to detect LDs that occurred at earlier stages. LD was determined according to the FIGO guidelines for fetal monitoring during delivery (Ayres-de-Campos et al., 2015). For the primary judgment, possible LDs were selected from a total of 552 cases by a midwife with 3 years of clinical experience under the supervision of expert obstetricians. As a final decision, cases of LD were extracted through agreement between two expert obstetricians (with 15 and 6 years of clinical experience, respectively). The data were classified with LD, without LD (hereafter referred to as non-LD), and LD-like signals (hereinafter referred to as LD-like). We defined LD based on FIGO guidelines, which are as follows, 1) A deceleration with an amplitude of 15 bpm or more that takes 30 s or longer to reach its nadir, 2) When uterine contractions are adequately recorded, LD is characterized by a decrease in heart rate that begins more than 20 s after the onset of a uterine contraction and returns to the baseline after the contraction ends, and 3) The morphological characteristics of FHR include a “deceleration with a gradual onset, gradual return to baseline, and/or reduced variability within the deceleration.” However, Some LDs are challenging to distinguish from prolonged deceleration (PD); however, PD is defined by the FIGO guidelines as a deceleration that lasts longer than 3 min. Thus, the duration of deceleration was also considered in the determination process. We defined the LD-like pattern as the signal in which the quality of the UC signal is poor and unreadable, but the FHR signal shows a gradual decline and recovery with a decrease in variability. Hence, LD-like patterns were positioned as possible LD signals. The data length used for analysis was 3 min per case (LD and LD-like: 1.5 min before and after the nadir point of FHR as the base point; non-LD: 3 min randomly selected from the total recording time, avoiding the interval where the signal loss was 100%).

### 2.2 Methods

**FIGURE 1:**
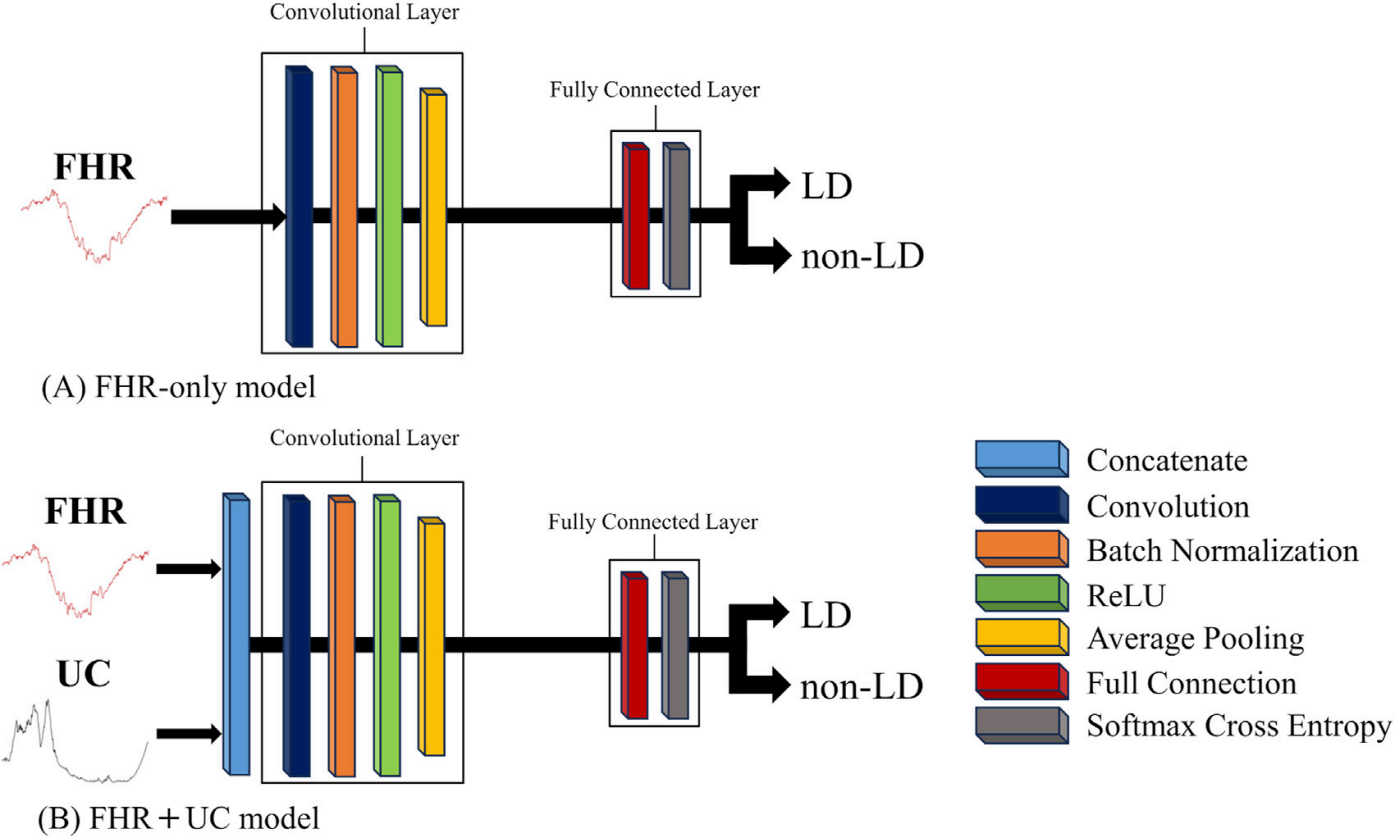
The basic structure of each AI model. The convolution and fully connected layer in each AI model is shown. **(A)** The FHR-only model employs a 1D-CNN, and **(B)** the FHR + UC model is constructed by concatenating FHR and UC, which are 1D signals, into a 2D signal to form a CNN model.

We used a CNNs to construct an FHR-only model to identify LD from FHR-only and an FHR + UC model to identify LD from FHR and UC. In the present study, we examined two LD classifications: LD and non-LD. A Neural network console was used to construct the AI models (Sony Neural Network, 2024). Figure 1 shows the basic structure of each model. The architecture of each model consisted of a convolutional layer, Batch Normalization, and a Rectified Linear Unit (ReLU) for the activation function. The output layer used a loss function with a softmax activation function (softmax cross entropy), and the class probabilities were generated by the classification task.

We used cross-validation to analyze the database because of a lack of LD data.

The FHR-only and FHR + UC models were evaluated for the binary classification of LD and non-LD. For the overall model accuracy, a receiver operating characteristic (ROC) curve was generated, and the area under the curve (AUC) was calculated. The results are classified into four categories: true negative (TN), false negative (FN), true positive (TP), and false positive (FP). Using these categories, the F-measure calculated from the precision and recall of LD, was used to compare the accuracy of the LD detection.

Precision (Equation 1) represents the percentage of samples that the model predicted to be LD that were actually LD, and it assesses the impact of false positives.

$$\text{Precision}=\frac{\text{TP}}{\text{TP}+\text{FP}}$$ (1)

Recall (Equation 2) indicates the percentage of samples for which the model correctly predicted LD and assessed the impact of false negatives.

$$\text{Recall}=\frac{\text{TP}}{\text{TP}+\text{FN}}$$ (2)

The F-measure (Equation 3) is an evaluation measure for binary classification tasks and an important indicator for assessing the ability to detect LD. In this study, we explored the architecture that would provide the highest F- measure in the case of LD by varying the number of convolutional layers, kernel parameters, batch size, and learning rate in the construction of the AI model.

$$F-\text{Measure}=\frac{2\times Precision\times Recall}{Precision+Recall}$$ (3)

Additionally, the output values of the AI were defined as the value representing the certainty of LD, and the paired-sample *t*-test was performed to calculate the 95% confidence intervals.

In clinical practice, obstetricians focus on changes in the FHR signal patterns to assess the condition of the fetus when the quality of the UC signal is poor. Previous studies have pointed out that UC signals were not of sufficient quality in the database used in previous studies (Xiao et al., 2022; Zeng et al., 2021). Therefore, we used the model with the highest accuracy (with or without UC) to infer cases from this database that were judged as LD-like by two obstetricians and gynecologists and confirmed the model’s judgment.

## 3 Results

**FIGURE 2:**
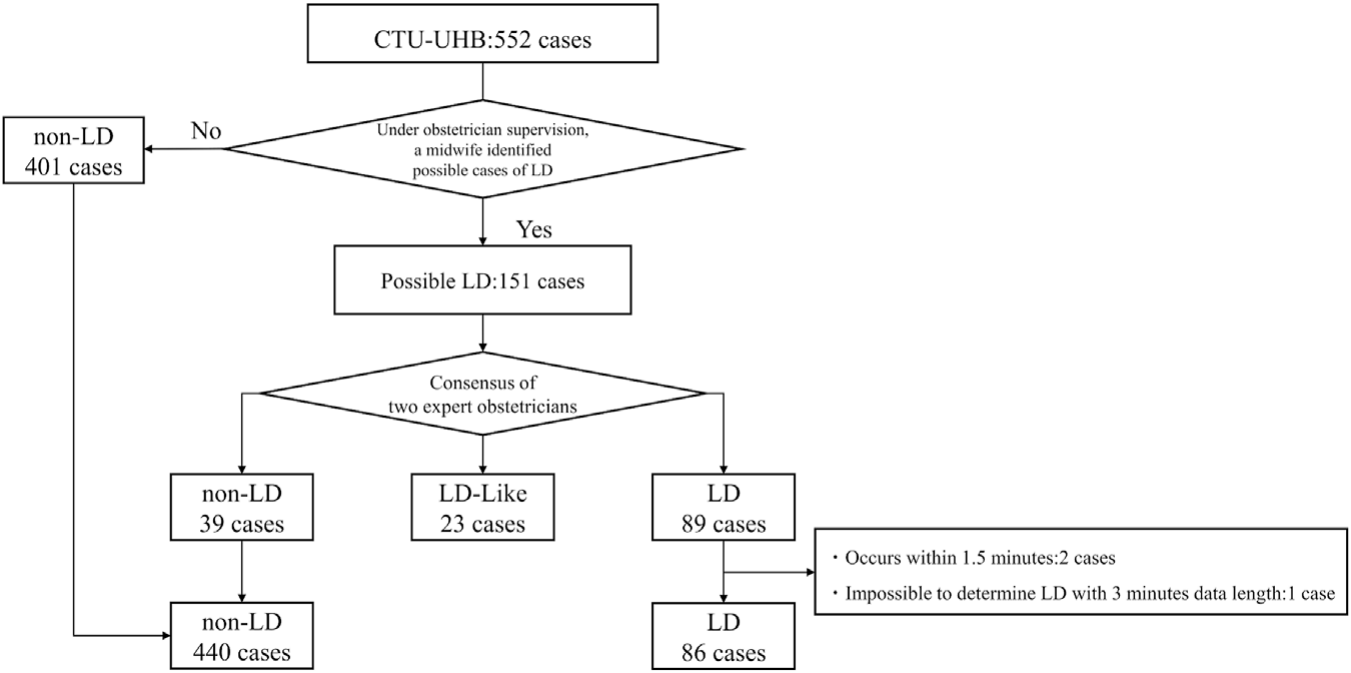
Flowchart of ground-truth labeling and data selection from the database. The process through which the 552 cases were classified into three classes is shown.

Figure 2 shows a flowchart for the ground-truth labeling and data selection. Two cases were excluded because the time from the start of recording to the onset of LD was too short to allow for data length, and one case was excluded because it was difficult to determine the LD within the data length determined in this study. As a result of the labeling and data selection, the final data used for analysis included 86 cases of LD, 440 cases of non-LD, and 23 cases of LD-like. To evaluate the generalization performance of the model, a 10-fold cross-validation was performed. For each dataset, the cross-validation, training, and validation datasets had a 9:1 ratio. Table 2 shows the numbers of LD and non-LD cases per fold. Due to a lack of the number of LD data, the data were augmented five times by shifting the data 30 s before and after the nadir point of the FHR and 15 s each. This made the number of LD and non-LD cases almost equal. By adjusting the detection accuracy to the highest value for each model, the FHR-only model showed the highest accuracy with four convolutional layers, and the FHR + UC model showed the highest accuracy with six convolutional layers. The kernel size was 1 × 13 for the FHR-only model and 1 × 17 for the FHR + UC model. The learning rate and the batch size for both models were 0.01 and 32, respectively.

**TABLE 2:** Number of cases with LD and non-LD in 10-fold. In each data set, the same cases were not included in the training and validation sets.

	Fold 1	Fold 2	Fold 3	Fold 4	Fold 5	Fold 6	Fold 7	Fold 8	Fold 9	Fold 10
LD	9	9	9	9	9	9	8	8	8	8
non-LD	44	44	44	44	44	44	44	44	44	44

**FIGURE 3:**
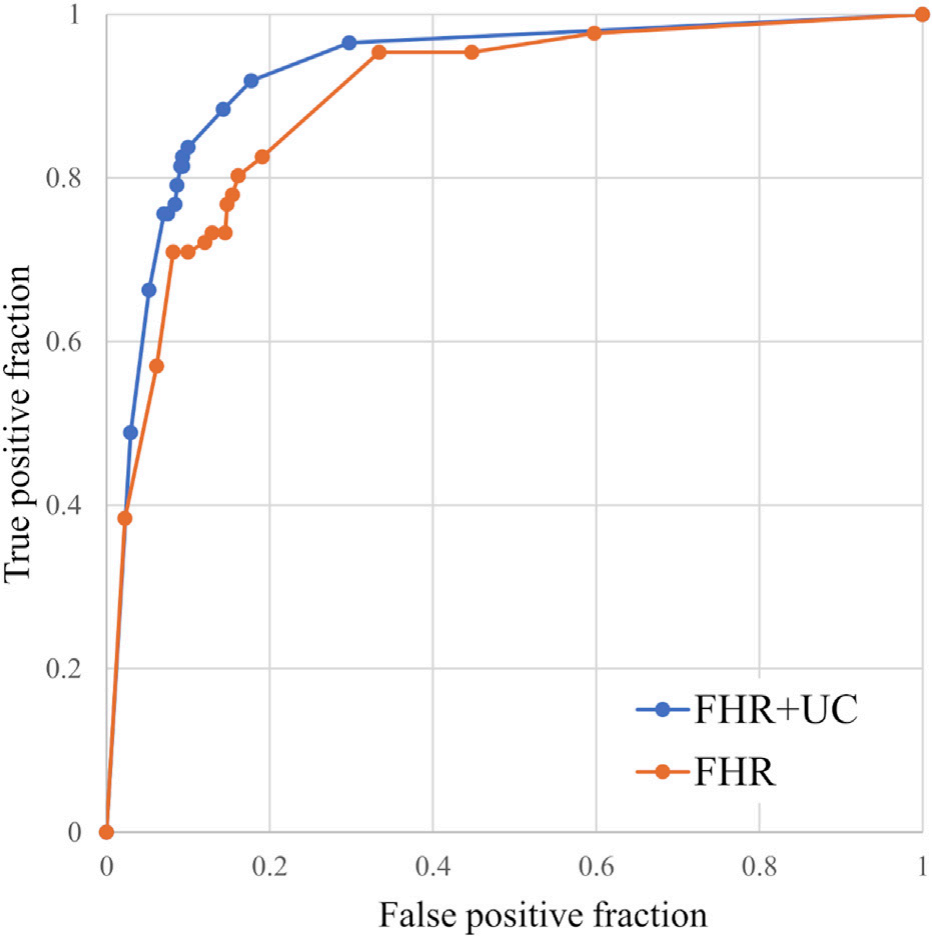
Comparison of ROC curves for the models with and without UC. We compared the classification accuracy of the LD and non-LD categories. The FHR-only model was also able to classify the LD and non-LD.

**FIGURE 4:**
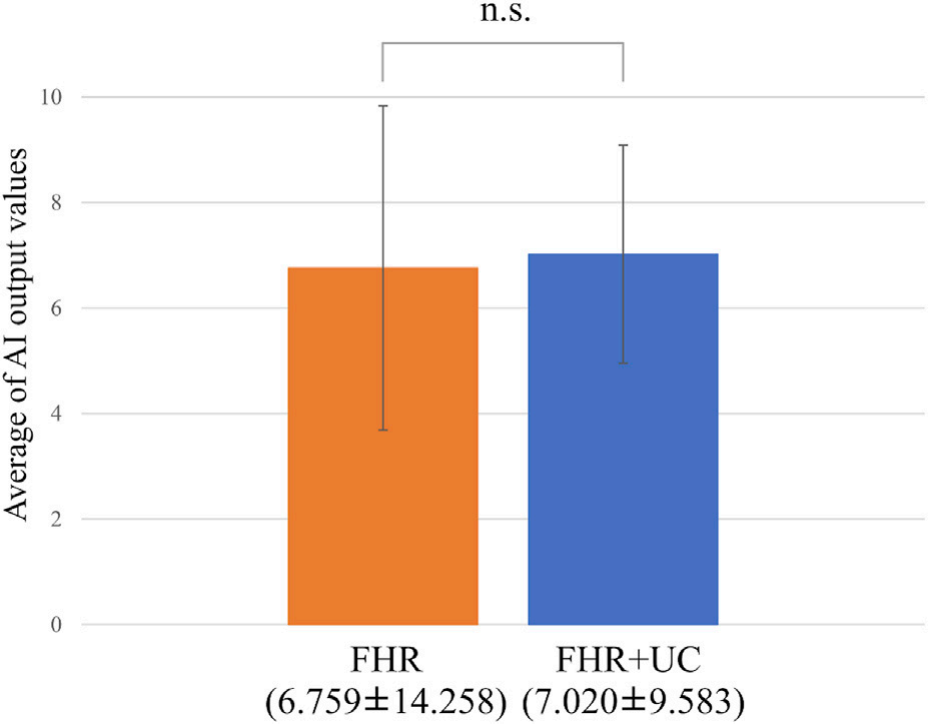
Comparing the probability of LD judgments. The vertical axis shows the average value of the AI output, and the error bar shows the 95% confidence interval. The average value test did not reveal any statistically significant differences.

Figure 3 summarizes the LD and non-LD classification accuracies in the FHR-only and FHR + UC models. Table 3 shows the F-measure for the LD and AUC for each model. The AUC and F-measure for LD were 0.896 and 0.613, respectively, for the FHR-only model and 0.928 and 0.711, respectively, for the FHR + UC model. Figure 4 shows the average output values of each model, with no statistically significant differences in the paired-samples *t*-test and overlapping error bars in the 95% confidence intervals of both models. Therefore, although the FHR-only model was less accurate than the FHR + UC model, there was no significant difference in the accuracy of LD detection, indicating that the FHR-only model also showed good results.

**TABLE 3:** Comparison of F-measure for AUC and LD for each model. Even with the FHR-only model, the detection accuracy of LD is high.

	AUC	F-measure
FHR	0.896	0.613
FHR + UC	0.928	0.711

**FIGURE 5:**
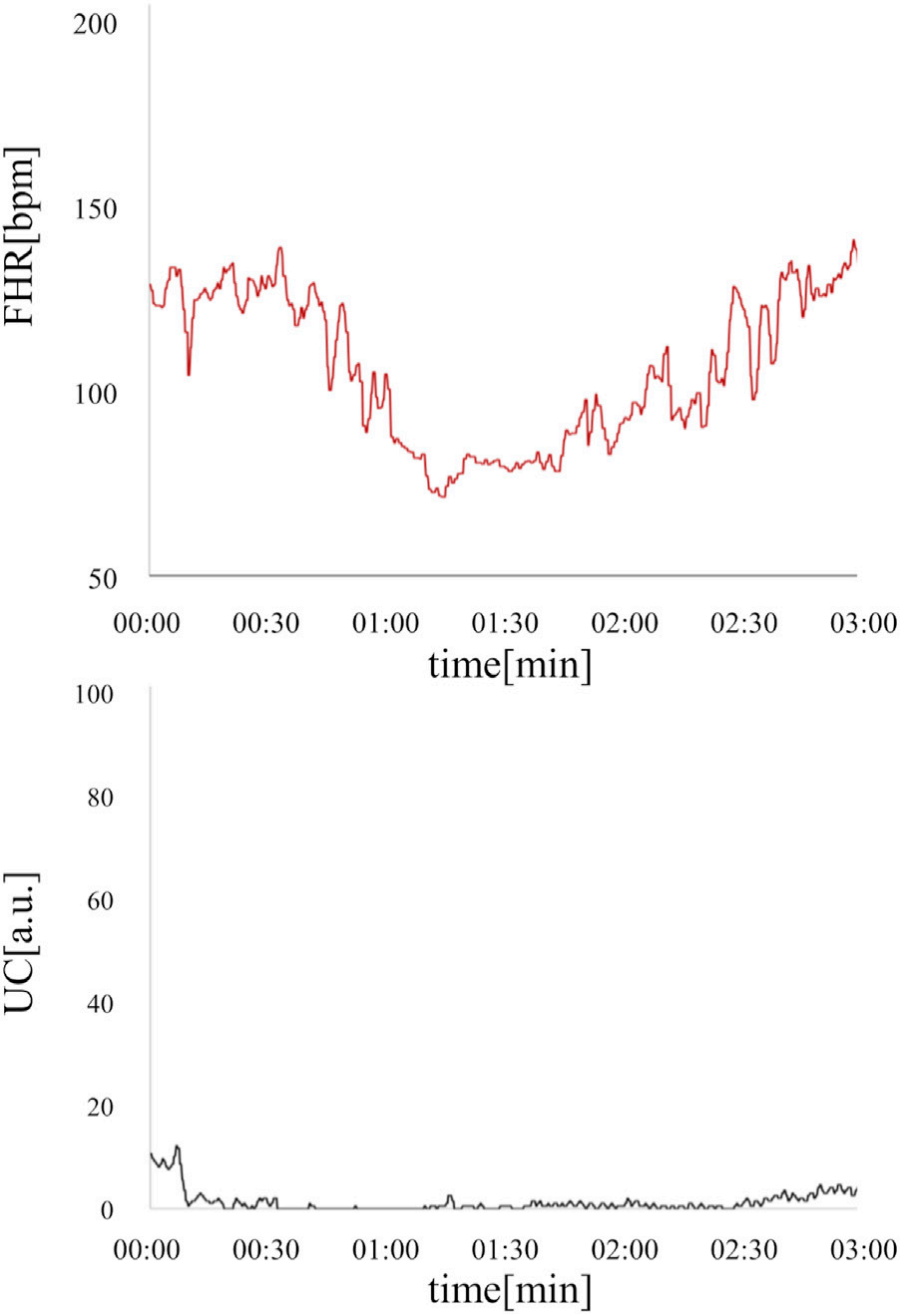
An example of an LD-like waveform is where UC is defective, but FHR takes the form of LD. This deceleration resembled PD, but was determined to be LD-like by a specialist obstetrician because the duration of the deceleration was less than 3 min and the UC signal was poor. The fetal heart rate is shown in red, and uterine contractions are shown in black.

**FIGURE 6:**
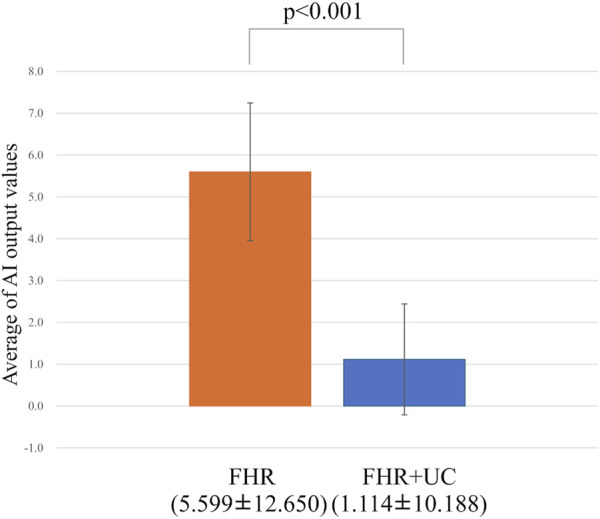
Comparing the probability of LD-like judgments. The vertical axis shows the average value of the AI output, and the error bar shows the 95% confidence interval. In the test of the average value, the FHR-only model had a statistically significant higher detection accuracy for LD-like than the FHR + UC model.

**FIGURE 7:**
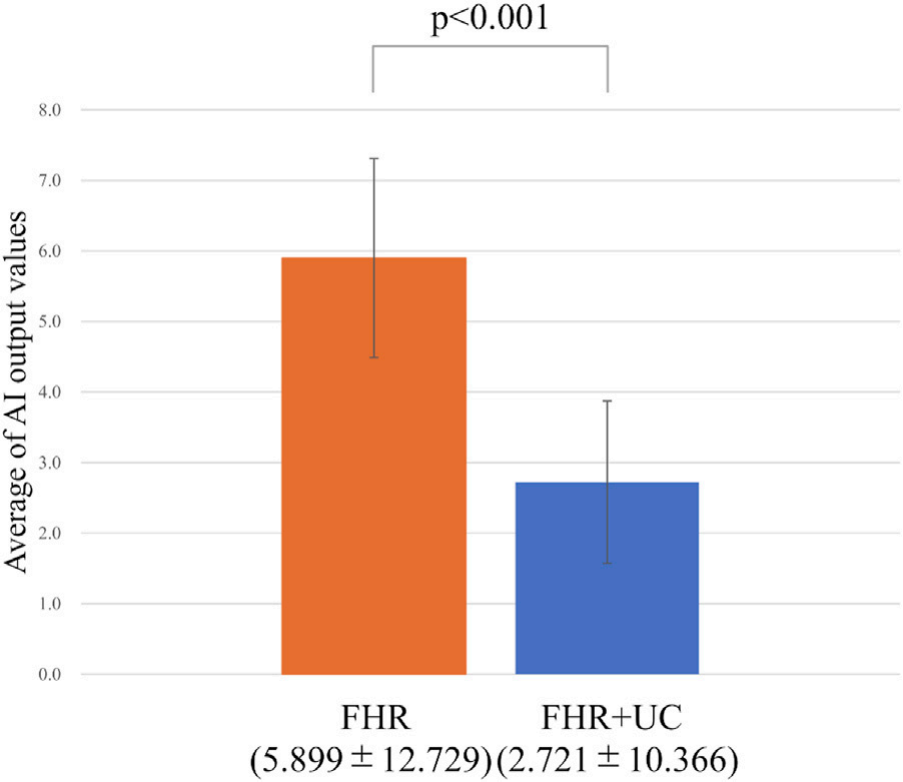
Comparing the probability of LD (including LD-like) judgments. The vertical axis shows the average value of the AI output, and the error bar shows the 95% confidence interval. In the test of the average value, the FHR-only model had a higher detection accuracy for LD (including LD-like) than the FHR + UC model.

**FIGURE 8:**
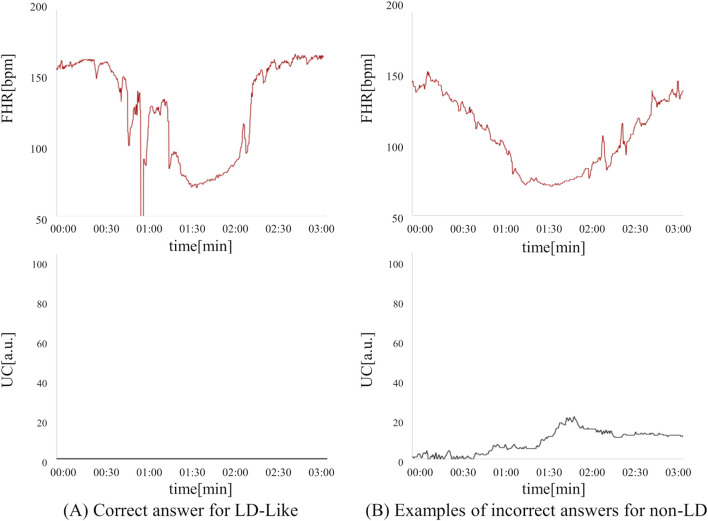
Comparison of correct answers for LD-like cases and incorrect answers for non-LD cases in the FHR-only model. **(A)** LD-like can be detected as a possible LD based on the morphological characteristics of FHR. **(B)** This is an example of a non-LD being mistakenly detected as an LD. This case is a PD case that did not recover to baseline within 3 min.

In the present ground-truth labeling, two expert obstetricians judged 4.2% (23/552 cases) of cases as LD-like. An example of a CTG waveform determined as an LD-like signal is shown in Figure 5. Table 4 and Figure 6 show the results of the validation using LD-like and non-LD cases. The FHR-only model yielded AUC and F-measures of 0.833 and 0.726, respectively, both of which were more accurate than the FHR + UC model. The 95% confidence interval error bars of the two models did not overlap in terms of the likelihood of LD, and the paired-sample *t*-test results showed that the FHR-only model was statistically superior in detecting LD-like cases (p < 0.001). Table 5 and Figure 7 present the validation results when LD-like was assumed to be LD and added to the LD cases. The FHR-only model yielded AUC and F-measure values of 0.867 and 0.747, respectively, both of which were more accurate than those of the FHR + UC model. The 95% confidence interval error bars of the two models did not overlap with the likelihood of LD, and the paired-sample *t*-test results showed that the FHR-only model was statistically superior in detecting LD-like cases (p < 0.001). Figure 8 shows case (A), which was most reliably detected as LD among the LD-like cases in the FHR-only model, which was highly accurate in detecting LD and LD-like cases, and case (B), in which a non-LD case was incorrectly detected as LD. Case (A) was highly accurate in detecting LD, and the morphological features of the FHR indicated that it could detect potential LD.

**TABLE 4:** Comparison of AUC and F-measure for LD-like for each model. The FHR-only model has a higher detection accuracy for LD-like.

	AUC	F-measure
FHR	0.833	0.726
FHR + UC	0.823	0.641

**TABLE 5:** Comparison of F-measure for AUC and LD (including LD-like) for each model. The FHR-only model has a higher detection accuracy for LD (including LD-like).

	AUC	F-measure
FHR	0.867	0.747
FHR + UC	0.851	0.713

## 4 Discussion

In this study, LDs were detected with high accuracy even in a model that used only the FHR signal. Therefore, the CNN constructed in this study could detect LD by capturing the baseline level of the FHR signal and its variability in acceleration or deceleration (Czabanski et al., 2012). The detection of LD using only the FHR signal may aid early, unmissable capture of signs of nonreassuring fetal status, even when the UC signal quality is poor. Additionally, inter- and intra-observer decipherment errors have been noted in the visual evaluation of CTG (Hernandez Engelhart et al., 2023; Rei et al., 2016); if the quality of the UC signal is poor, the difficulty in deciphering UC may lead to greater decipherment errors. Therefore, detecting LD with high accuracy and providing alerts based on FHR signals alone better supports CTG decipherment. Furthermore, alerting obstetric healthcare providers to signs of nonreassuring fetal status may encourage them to reattach sensors that measure pressure changes during uterine contractions. Reattachment of the sensor is of great clinical significance because it enables subsequent CTG readings to evaluate fetal health status in consideration of the temporal relationship between FHR and UC.

The results of the inference using the LD-like case showed that the FHR-only model could detect potential LD signals with high accuracy. Since it is important not to miss an LD, an LD-like signal should be detected as a potential LD signal. Thus, assuming that LD-like cases are indeed signs of LD and UC could not be detected, alerting them using the FHR-only model may lead to earlier detection of LD, the early marker of fetal mild hypoxemia.

In this study, we tested whether LD, the early marker of fetal mild hypoxemia, could be detected from only the FHR signal. However, specificity needs to be considered for clinical use. Particularly, since it is important to discriminate cases of variable deceleration (VD) (Japan Council for Quality Health Care, 2018), which is considered difficult to discriminate from LD and other decelerations, we conducted an evaluation using cases with VD. Of the non-LD cases in the database, 4.8% (21/440) were determined to have VD by the consensus of expert obstetricians at the time of final determination. The F-measure and percentage of correct answers for VD cases in each model were 0.822% and 66.6% (14/21), respectively, for the FHR-only model and 0.680% and 47.6% (10/21), respectively, for the FHR + UC model. The FHR-only model achieved higher accuracy in terms of both F-value and correct response rate. This suggests that the LD and VD could be detected with high accuracy using only the FHR signal. The differences in the morphological characteristics of the FHR in cases of the LD and VD are that the VD shows a rapid drop (onset to nadir in less than 30 s), good variability within the deceleration, and rapid recovery to baseline (Ayres-de-Campos et al., 2015). This suggests that the LD and VD could be correctly distinguished by capturing the morphological characteristics of the FHR. VD also accounts for the majority of bradycardia that occurs during labor and is considered a baroreceptor-mediated response to elevated arterial pressure, such as that occurring with cord compression (Ball and Parer, 1992). In other words, since VD is often a physiological response of the fetus during the delivery period, it is beneficial to distinguish it from LD, which is a sign of fetal hypoxemia.

Subsequently, 440 non-LD cases were analyzed. In 14.7% (65/440) of false positive cases, non-LD cases were misclassified as LD. Typically, 33 cases of intermittent interruption of the FHR signal were included. Additionally, three false positive cases were very close to the signal pattern of LDs. Specifically, in the case (B) shown in Figure 8, two expert obstetricians determined that the patient had PD because the recovery from the heart rate drop to baseline was only slightly longer than 3 min. Since PD is also a sign of fetal hypoxemia (Chandraharan and Arulkumaran, 2007), we believe that it is worthwhile to alert the observer in the same manner as that in LD; therefore, we do not see a major problem with false positives in these cases.

This study has three limitations. First, we used a simple CNN configuration model because the dataset was relatively small. After increasing the amount of data, we expect to obtain better results using the latest models. Second, we used fragmented data. It is necessary to evaluate this by constructing a system that can discriminate LD in real-time using continuous data. Third, we limited the target of detection to the first LD; however, we believe that it is important to detect LD as early as possible to ensure early medical intervention. However, it is possible to improve the detection accuracy of LDs if we use all the LDs in the dataset, not just the first.

This comparison and verification of the accuracy of LD detection with and without UC signals using a CNN showed that LD could be detected with high accuracy from the FHR signal only. This result suggests that the LD could be detected using the FHR signal, even when the quality of the UC signal is poor. Alerting obstetric healthcare providers to signs of nonreassuring fetal status will encourage them to closely monitor and prepare for rapid delivery, which is expected to improve fetal outcomes.

## Data Availability

The original contributions presented in the study are included in the article/supplementary material, further inquiries can be directed to the corresponding author.

## Abbreviations

CTG, Cardiotocography; FHR, Fetal heart rate; UC, Uterine contraction; LD, Late deceleration; AI, Artificial Intelligence; CNN, Convolutional Neural Network; AUC, Area under the curve; FIGO, International Federation of Gynecology and Obstetrics; ML, Machine learning; DL, Deep learning; ReLU, Rectified Linear Unit; ROC, Receiver Operating Characteristic; TN, True negative; FN, False negative; TP, True positive; FP, False positive; VD, variable deceleration; PD, prolonged deceleration.
